# Structural optimization of reversible dibromomaleimide peptide stapling

**DOI:** 10.1002/pep2.24157

**Published:** 2020-03-20

**Authors:** Ayanna Lindsey‐Crosthwait, Diana Rodriguez‐Lema, Martin Walko, Christopher M. Pask, Andrew J. Wilson

**Affiliations:** ^1^ School of Chemistry, University of Leeds Leeds UK; ^2^ Astbury Centre for Structural Molecular Biology, University of Leeds Leeds UK

**Keywords:** constrained peptides, protein‐protein interactions, dibromomaleimide, peptide conformation

## Abstract

Methods to constrain peptides in a bioactive α‐helical conformation for inhibition of protein‐protein interactions represent an ongoing area of investigation in chemical biology. Recently, the first example of a reversible “stapling” methodology was described which exploits native cysteine or homocysteine residues spaced at the *i* and *i* + 4 positions in a peptide sequence together with the thiol selective reactivity of dibromomaleimides (a previous study). This manuscript reports on the optimization of the maleimide based constraint, focusing on the kinetics of macrocyclization and the extent to which helicity is promoted with different thiol containing amino acids. The study identified an optimal stapling combination of *X*_1_ = L‐Cys and *X*_5_ = L‐*h*Cys in the context of the model peptide Ac‐X_1_AAAX_5_‐NH_2_, which should prove useful in implementing the dibromomaleimide stapling strategy in peptidomimetic ligand discovery programmes.

## Graphical abstract

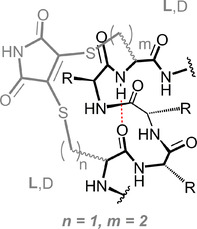

## 1 INTRODUCTION

**FIGURE 1:**
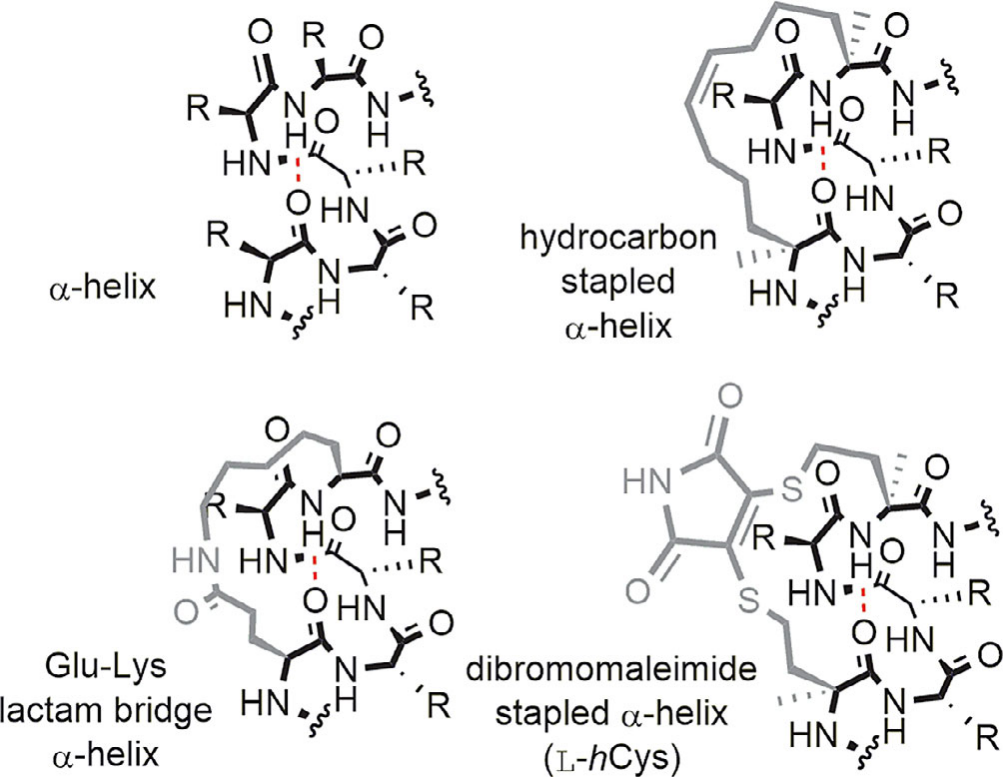
Schematic structures for an α‐helix together with representative constraints for *i* to *i* + 4 residues; the widely employed hydrocarbon constraint, the most effective helix inducing lactam constraint, and the dibromomaleimide constraint explored in this study

The development of methodology to constrain peptides in a bioactive conformation for the purposes of inhibiting protein‐protein interactions represents an area of significant effort.^[^ ^1^, ^2^, ^3^, ^4^, ^5^, ^6^, ^7^, ^8^, ^9^, ^10^, ^11^, ^12^, ^13^ ^]^ Widely applied to the pre‐organization of helical epitopes and popularized as “stapling”, the introduction of a constraint between the *i* and *i* + 4 residues (Figure 1)—or to a lesser extent, *i* + 7 or *i* + 11 residues—in a peptide sequence has been shown to be effective in biasing peptides towards a helical conformation.^[^ ^2^, ^14^, ^15^, ^16^, ^17^, ^18^, ^19^, ^20^, ^21^, ^22^, ^23^, ^24^, ^25^, ^26^, ^27^, ^28^, ^29^, ^30^ ^]^ This pre‐organization can lead to improved binding potency towards target, increased (proteolytic) stability and increased cell permeability.^[^ ^1^, ^3^, ^31^, ^32^, ^33^, ^34^, ^35^, ^36^ ^]^ Despite this progress, there remains a need to develop new synthetic methodology for constraining peptides that is readily implemented by non‐specialists, and which can be applied without recourse to sequences bearing specialized amino acids. Key factors that determine the effectiveness with which introduction of a constraint biases the peptide conformation towards an α‐helix include: the length and rigidity of tether between the covalently linked amino acids; the amino acid stereochemistry; the presence of stereogenic centres within the tether and, steric, electrostatic or dipolar interactions between functionality in the tether and the peptide sequence.^[^ ^37^, ^38^, ^39^ ^]^

Our group recently introduced the first reversible stapling methodology,^[^ ^25^ ^]^ which exploits native cysteine or homocysteine residues spaced at the *i* and *i* + 4 positions in a peptide sequence together with the thiol selective reactivity of dibromomaleimides (Figure 1).^[^ ^40^, ^41^, ^42^, ^43^ ^]^ Using *N*‐Alkynyl‐dibromomaleimides permits further functionalization through “click” chemistry allowing the facile constraint and diversification of readily and/or commercially available thiol containing peptides. The widely applied hydrocarbon stapling introduced by Verdine that relies on intramolecular ring closing metathesis of judiciously placed *S*‐pentenylalanine residues served as inspiration for our original design. To be *iso*atomic with the hydrocarbon constraint we used homocysteine to generate constrained peptides of BID (a BH3 only effector member of the BCL‐2 family) and RNase S peptide to interact with (anti‐apoptotic members of) the BCL‐2 family and RNase S protein (a widely studied enzyme which cleaves RNA) respectively. Although, the homocysteine variants were generally more effective than the cysteine variants in promoting a helical conformation and conferring enhanced affinity/ function to either sequence, the later were also clearly tolerated. Moreover, cyclization of the cysteine variants proceeded more rapidly. These observations motivated a more detailed sequence‐structure analysis which we describe in the current manuscript; we show that whilst the rate of cyclisation is independent of the length of the constraint, the optimal stapling combination in the context of the model peptide Ac‐X_1_AAAX_5_‐NH_2_ employs *X*_1_ = L‐Cys and *X*_5_ = L‐*h*Cys.

## 2 MATERIALS AND METHODS

All reagents were purchased from either Sigma‐Aldrich, Acros Organics or Fluorochem and were used without further purification. Solvents used for reactions and workups were purchased from Fisher Chemical (HPLC grade). Deionized and milliQ water were obtained from an Elga Water Purification system. All amino acids were *N*‐Fmoc protected and side chains were protected with; Trt (Cys, hCys); Mtt (Lys) and OPip (Asp). The evaporation of solvents was achieved using a Büchi R3 with a Vacubrand CVC3000 vacuum pump and condenser connected to a recirculating cooler system Julabo F1000. LC‐MS analyses were conducted on a ThermoScientific Dionex UltiMate 3000 and high‐resolution mass spectrometry (HR‐MS) data were recorded using electrospray ionization in positive mode (ESI+) with a Bruker MaXis Impact spectrometer. Preparative HPLC experiments were performed using an Agilent 1260 Infinity instrument with a Jupiter Proteo 90 Å 250 × 21.2 mm, 10 μm preparative column. Analytical HPLC experiments were performed using an Agilent 1290 Infinity LC series system equipped with an Ascentis Express Peptide ES‐C18 100 × 2.1 mm column, 2.7 μm particle size on a 5%‐50% gradient of acetonitrile in water (with 0.1% formic acid) over 10 min.

### 2.1 Solid‐phase peptide synthesis

Linear pentapeptides (0.25 mmol, 1 eq) were synthesized on Rink amide resin (loading capacity 0.81 mmol/g) using DIC and Oxyma in DMF for amide coupling, 10% acetic anhydride in DMF for the acetylation of the *N*‐terminus and 20% piperidine in DMF for the deprotection. Standard methods of coupling and deprotection supplied with CEM Liberty Blue peptide synthesizer with microwave assistance were used. After the synthesis, the resin was drained, washed with DMF (2 × 2 mL × 2 min), DCM (2 × 2 mL × 2 minutes) and diethyl ether (3 × 2 mL × 2 min) and dried for 1 h. Protecting groups and the peptide were simultaneously cleaved from the resin with TFA: H_2_O: TIPS: EDT (92.5:2.5:2.5:2.5; 5 mL × 3 h). The resin was then washed with fresh TFA (2 mL × 2 min × 2). The solvent was finally evaporated under nitrogen flux and the resulting oils were dissolved in dioxane: water (1:1) and freeze dried. Peptides **1a‐g** were then purified using preparative HPLC. Fractions were checked using LC‐MS, and fractions containing the target peptide were concentrated on the rotary evaporator and freeze dried.

**Table:** 

Peptide	*m/z* [1H]^+^ found	*m/z* [1H]^+^ calcd.	Formula
**1a**	479.1750	479.1747	C_17_H_31_N_6_O_6_S_2_
**1b**	479.1744	479.1747	C_17_H_31_N_6_O_6_S_2_
**1c**	479.1739	479.1747	C_17_H_31_N_6_O_6_S_2_
**1d**	479.1514	479.1747	C_17_H_31_N_6_O_6_S_2_
**1e**	493.1901	493.1903	C_18_H_33_N_6_O_6_S_2_
**1f**	493.1903	493.1898	C_18_H_33_N_6_O_6_S_2_
**1g**	507.2063	507.2054	C_19_H_35_N_6_O_6_S_2_

Peptide **1a** (14.2 mg, 0.03 mmol, 12%), Peptide **1b** (4.2 mg, 8.8 μmol, 4%), Peptide **1c** (6.5 mg, 14 μmol, 5%), Peptide **1d** (3.7 mg, 7.7 μmol, 3%), Peptide **1e** (9.2 mg, 18 μmol, 7%), Peptide **1f** (14 mg, 14%), Peptide **1g** (8.5 mg, 9%); (see Supporting Information for HPLC traces and HRMS, Figures S1 and S2).

### 2.2 Preparation of constrained peptides 1‐2

The syntheses of precursor crude acetylated linear peptides (0.25 mmol, 1 eq) was carried out as described above and these were used in the following stapling reaction without further purification.

Peptide solutions (0.1 mmol) in a mixture of acetonitrile: 50 mM ammonium acetate buffer pH 7.4 (1:1, 30 mL) were treated with TCEP (32 mg, 0.11 mmol) and the reaction mixtures were stirred at room temperature. Peptides were shown by LC‐MS to be completely reduced after 1 h. 2,3‐Dibromomaleimide (28 mg, 0.11 mmol) was then added and the reactions stirred at room temperature for a further 1 h. After confirming the reactions were complete by LC‐MS the reaction mixtures were freeze dried. Crude peptides were dissolved in 5 mL of methanol and purified using preparative HPLC. Peptides were purified using a flow rate of 20 mL/min and a gradient of 20‐40% methanol in water containing 0.1% TFA.

**Table:** 

Peptide	*m/z* [1H]^+^ found	*m/z* [1H]^+^ calcd.	Formula
**2a**	572.1591	572.1597	C_21_H_30_N_7_O_8_S_2_
**2b**	572.1588	572.1597	C_21_H_30_N_7_O_8_S_2_
**2c**	572.1578	572.1597	C_21_H_30_N_7_O_8_S_2_
**2d**	572.1589	572.1597	C_21_H_30_N_7_O_8_S_2_
**2e**	586.1745	586.1748	C_22_H_32_N_7_O_8_S_2_
**2f**	586.1753	586.1748	C_22_H_32_N_7_O_8_S_2_
**2g**	600.1902	600.1905	C_23_H_34_N_7_O_8_S_2_

Peptide **2a** (14.2 mg, 25 μmol 21%), Peptide **2b** (4.2 mg, 7.3 μmol, 7%), Peptide **2c** (12 mg 20 μmol, 21%), Peptide **2d** (6.0 mg, 10 μmol, 11%), Peptide **2e** (2.2 mg, 3.7 μmol, 4%); Peptide **2f** (10.8 mg, 18.4 μmol, 18%); Peptide **2g** (9.9 mg, 16.5 μmol, 17%); (see Supporting Information for HPLC traces and HRMS Figures S1 and S3).

### 2.3 Synthesis of peptide 3

*N*‐Fmoc amino acids had chains protected with Mtt (Lys); OPip (Asp). Ac‐K(Mtt)AAAD(OPip)‐OH (0.1 mmol, 1 eq) was synthesized on Rink amide resin (loading capacity 0.123 mmol/g) using DIC and Oxyma in DMF for amide coupling, 10% acetic anhydride in DMF for the acetylation and 20% piperidine in DMF for the deprotection using a CEM Liberty Blue peptide synthesizer with microwave assistance. The resin was then drained, washed with DCM (2 × 2 mL × 2mins), and treated repeatedly with 2% TFA in DCM (2 mL × 5 × 2 min). After washing with DMF (2 × 2 mL, 2mins), a solution of PyBOP (4 eq) and DIPEA (4 eq) in DMF was added to the resin and the reaction was agitated overnight. After the synthesis, the resin was drained, washed with DMF (2 × 2 mL × 2 min), DCM (2 × 2 mL × 2 min) and diethyl ether (3 × 2 mL × 2 min) and dried for 1 h. Subsequently, the protecting groups and the peptide were simultaneously cleaved from the resin with TFA: H_2_O: TIPS: EDT (92.5:2.5:2.5:2.5; 5 mL × 3 h). The resin was then washed with fresh TFA (2 mL × 2 min × 2). The solvent was finally evaporated under nitrogen flux and the resulting oils were dissolved in dioxane: water (1:1) and freeze dried. The peptide was then dissolved in acetonitrile and purified using preparative HPLC. The peptide (7.4 mg, 15 μmol 15%), eluted at a flow rate of 20 mL/min and a gradient of 0%‐40% acetonitrile in water (with 0.1% TFA); ESI HRMS found *m/z* 498.2680 [M + H]^+^, [C_21_H_36_N_7_O_7_]^+^ requires 498.2676 (see Supporting Information for HPLC trace and HRMS Figures S1 and S3).

### 2.4 UV absorbance analyses

UV absorbance analyses were recorded using a 10 mm path‐length quartz cuvette on a Cary 100 UV/VIS spectrometer. Peptide solutions (250 μM) were prepared from aqueous peptide stock solutions (10 mM phosphate buffer, pH 7.2) and samples were measured. Additionally, samples were also prepared in 50% TFE/10 mM phosphate buffer pH 7.2. The absorbance at 400 nm was recorded for peptides stapled with maleimide, whilst the absorbance at 205 nm was recorded for the lactam stapled peptide. The relative concentrations of the samples could then be calculated using the Beer‐Lambert law. Literature values for the absorption coefficient of maleimide^[^ ^25^ ^]^ and lactam^[^ ^38^, ^44^ ^]^ stapled peptides were used for the calculation.

### 2.5 Kinetic analyses

The kinetic mesurements were performed in 96 well‐plates using a Perkin Elmer EnVision 2103 Multilabel Reader. The 200 μL solution in each well contained 100 μM of peptide, 100 μM of TCEP and 200 μM of 2,3‐dibromomaleimide in 50 mM phosphate buffer pH = 6. The reactions were followed for 1 h at 20 °C.

### 2.6 Circular dichroism analyses

CD data were recorded using an Applied Photophysics Chirascan Instrument and Software in a 1 mm quartz cuvette. For each scan, the following parameters were used: 185‐260 nm range at 50 nm/ min, with a bandwidth of 1.0 nm, response time of 1 s and resolution step width of 1 nm. Each spectrum represents the average of five scans. The raw CD data obtained were processed by averaging the data for five scans and subsequently subtracting the solvent signal. Next, the CD values were converted into a relative mean residue ellipticity using the following equation:

$${\left[\theta \right]}_{215}={\left[\theta \right]}_{\mathrm{obs}}/\left(10*l*c\right)\left(n-1\right)$$

[θ]_215_ = observed mean residue ellipticity of a peptide at a given wavelength; [θ]_obs_ = measured ellipticity (mdeg); l = path length (cm); *c* = molar concentration (mol dm^−3^); *n* = number of peptide resides.

Percentage helicity (*f*_Helix_) was then calculated for peptides using the observed mean residue ellipticity of peptides at 215 nm and the following equation

$$f_{\text{Helix}}=\left[{\left[\theta \right]}_{215}\right]-\left[{\left[\theta \right]}_{0}\right]/\left[{\left[\theta \right]}_{\max}-{\left[\theta \right]}_{0}\right]$$

[θ]_0_ = mean residue ellipticity of the peptide in random coil formation (2220‐53 T); *T* = temperature (°C); [θ]_max_ = maximum theoretical mean residue ellipticity for a helix of n residues, (−44 000 + [250 *T*])*(1 − [*x*/*n*]) where *T* = temperature (°C); *x* = empirical constant, 3; *n* = number of peptide residues.

Finally, the percentage helicity was normalized relative to the percentage helicity of the lactam constrained peptide **3**, which was taken to have 100% α‐helicity.

### 2.7 X‐Ray analyses

A solution of **2a** (0.5 mg/mL) was dissolved in a mixture of water/ methanol (1:1) and following 2 days of slow evaporation at room temperature, the constrained peptide crystallized as yellow needles. X‐ray structure determination was carried out at 120 K on an Agilent SuperNova diffractometer equipped with an Atlas CCD detector and connected to an Oxford Cryostream low temperature device using mirror monochromated Cu Kα radiation (λ = 1.54184 Å) from a Microfocus X‐ray source. The structure was solved by direct methods using SHELXS^[^ ^38^ ^]^ and refined by a full matrix least squares technique based on F^2^ using SHELXL2014.^[^ ^39^ ^]^ The compound crystallized in a triclinic cell and was solved in the P1 space group, with one molecule and two molecules of water in the asymmetric unit. All non‐hydrogen atoms were located in the Fourier Map and refined anisotropically. All carbon‐bound hydrogen atoms were placed in calculated positions and refined isotropically using a “riding model.” All heteroatom bound hydrogen atoms were located in the Fourier Map and refined isotropically (see Supporting Information structure parameters Table S1).

## 3 RESULTS AND DISCUSSION

**Scheme 1:**
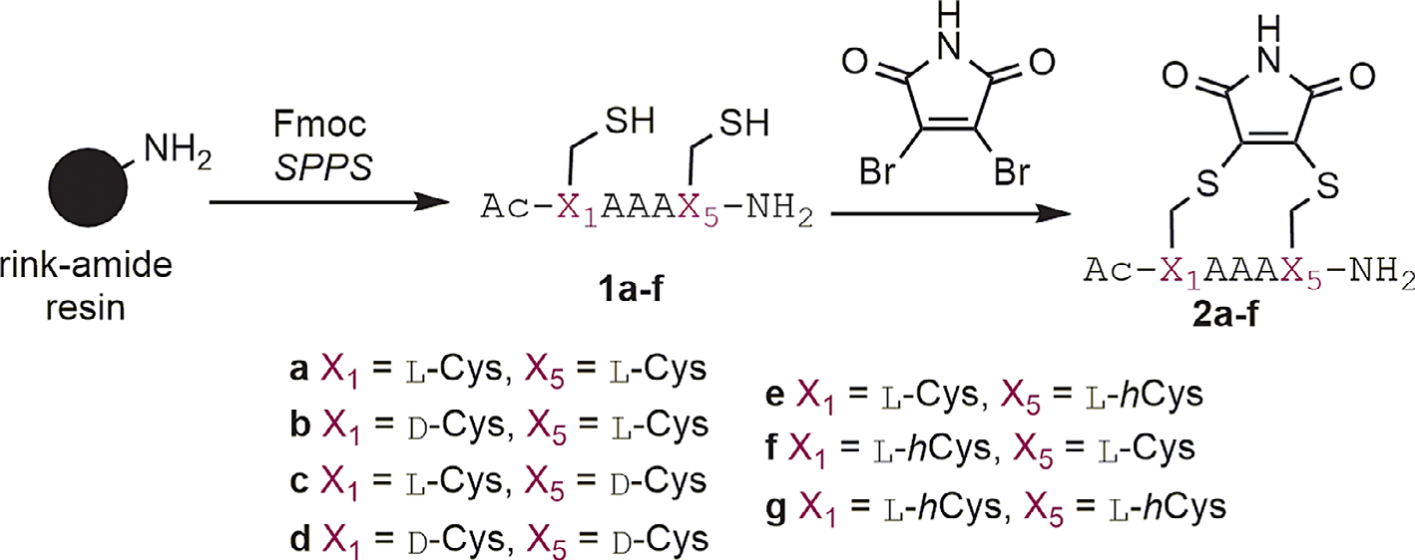
Synthesis of constrained pentapeptides **2a‐g**

We selected a pentameric alanine containing sequence as a model for these studies. This sequence has been widely used by Fairlie and co‐workers to compare different synthetic constraints.^[^ ^40^ ^]^ We prepared seven different peptides **1a‐g** bearing different combinations of D or L‐cysteine and L‐homocysteine at the *i* and *i* + 4 positions. These could readily be reacted with dibromomaleimide to generate the constrained peptides **2a‐g** (Scheme 1 and Table 1).

**TABLE 1:** Structures and helicities of constrained peptides

	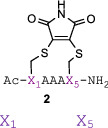		% Helicity 10 mM sodium phosphate at 215 nm	% Helicity 10 mM sodium phosphate (50% TFE) at 215 nm	% Helicity 10 mM sodium phosphate at 222 nm	% Helicity 10 mM sodium phosphate (50% TFE) at 222 nm
**a**	L‐Cys	L‐Cys	25	41	20	42
**b**	D‐Cys	L‐Cys	28	27	14	16
**c**	L‐Cys	D‐Cys	17	26	14	23
**d**	D‐Cys	D‐Cys	13	11	0	3
**e**	L‐Cys	L‐*h*Cys	48	66	40	56
**f**	L‐*h*Cys	L‐cys	11	32	6	29
**g**	L‐*h*Cys	L‐*h*Cys	13	30	9	25
**3**	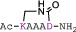		100	100	100	100

**FIGURE 2:**
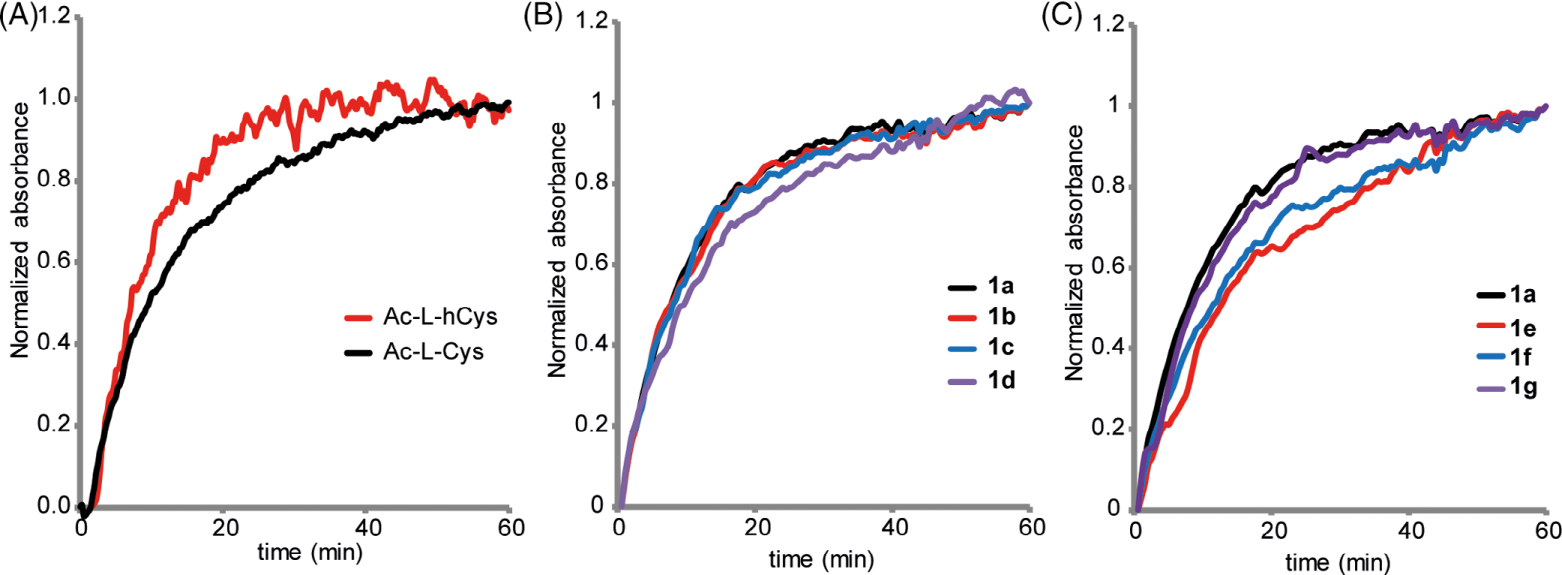
Kinetic analyses of stapling reactions (A) reaction of amino acids Ac‐L‐Cys (0.32 μM) and Ac‐L‐hCys (0.32 μM) with dibromomaleimide (0.16 μM) (50 mM, phosphate buffer pH 6.0), (B) reaction of **1a‐d** (100 μM) with dibromomaleimide and (C) reaction of **1a** and **1e‐g** (100 μM) with dibromomaleimide (200 μM) (50 mM, phosphate buffer pH 6.0, 20°C, λ = 395 nm)

To evaluate our prior qualitative observation that BID and RNase S peptide cysteine variants were more amenable to cyclization,^[^ ^25^ ^]^ we first compared the rate of reaction of suitably protected amino acids cysteine and homocysteine with dibromomaleimide. These analyses were conducted to allow us to dissect out any differences in the reactivity of the amino acid from differences in ring size for the cyclization; cysteine being anticipated to react more quickly due to the proximal α‐carbonyl. The reactions were followed by UV‐vis with the bromide for thiol substitution leading to a diagnostic spectroscopic change allowing a qualitative analysis of the reactivity. The analyses indicate that the cyclization proceeds significantly faster than that observed in our original study,^[^ ^25^ ^]^ although the greater length of the peptide is likely to influence the accessible conformations and side‐chains of amino acids proximal to the cysteine's will differentially affect the steric environment, and therefore, rate of cyclization, so caution should be exercised in directly comparing this work and the previous study. A mathematical analysis was hampered by competing hydrolysis of the maleimide ring,^[^ ^47^ ^]^ nonetheless the experiments indicated clearly only minor differences between the two amino acids (Figure 2A). Next the rates of cyclization of the peptides **1a‐d** were assessed. These indicated no dependence of the cyclization rate on amino acid stereochemistry, with all sequences generating near identical cyclization rates (Figure 2B). The rates of cyclization of peptides with one or both cysteine residues replaced with homocysteine **1e‐g** also showed a very small effect of the length of the created tether on the cyclization kinetics (Figure 2C).

**FIGURE 3:**
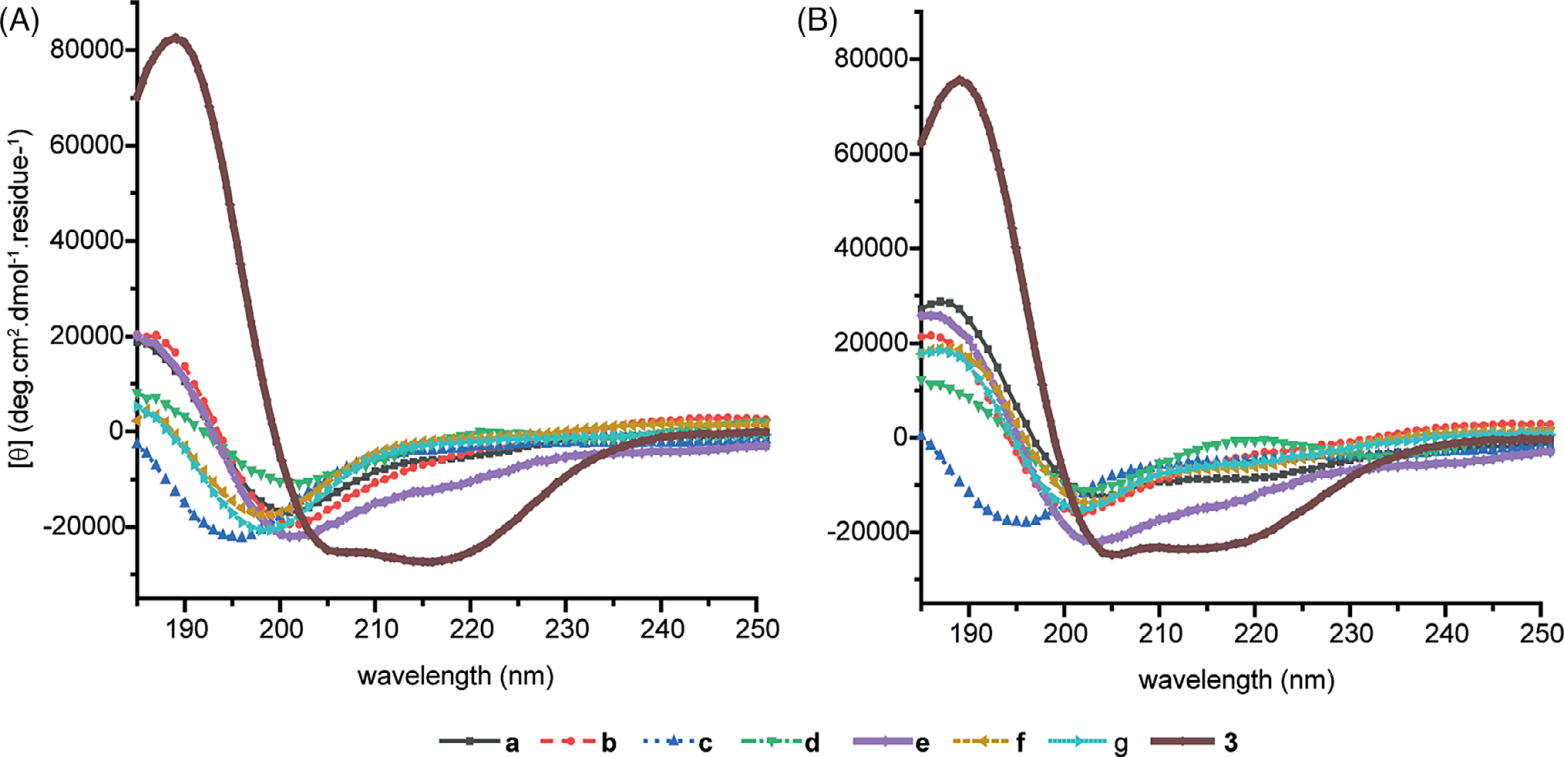
Circular Dichroism analyses of peptides **2a‐g** and **3** (A) peptide concentration 250 μM (10 mM phosphate buffer, pH 7.2, 298 K), (B) peptide concentration 250 μM (50% TFE, 10 mM phosphate buffer and pH 7.2, 298 K)

We then performed circular dichroism spectroscopy in phosphate buffer and phosphate buffer with 50% trifluoroethanol (TFE). To facilitate comparison, we also prepared the previously reported and optimally constrained sequence **3**, bearing a lactam bridge between Lys1 and Asp4 as a control;^[^ ^40^ ^]^ this lactam constraint was previously shown to be the most effective in a comparative study of *i*, *i* + 4 constraints leading to the maximum mean residue helicity for a five residue peptide. Our CD analysis indicated that peptide **2e** with *X*_1_ = L‐Cys and *X*_5_ = L‐*h*Cys to be superior to all other combinations in biasing the conformation of the peptides towards an α‐helical conformation (Figure 3 and Table 1). We used a wavelength of 215 nm (to calculate helicities as it has been shown that the minimum for shorter helices shifts to lower wavelength,^[^ ^48^, ^49^ ^]^ and this was the value used previously for the reference control lactam bridged **3**.^[^ ^40^ ^]^ Values at 222 nm are also given, however the trends are similar. Although not as effective as the lactam bridge, relative to **3** (100% helicity in buffer and 50% TFE), **2e** was observed to have helicity of 48% (buffer) and 66% (50% TFE). None of the remaining peptides **2a‐d** and **2f‐g** exhibited helicities more than two thirds of the values for **2e**, although more pronounced improvements on addition of TFE were observed in some instances. Whilst the increase in helicity on addition of the helix promoting solvent suggests the maleimide constraint is not fully optimal, the lower helicity of peptides **2a‐g** in comparison to **3** likely reflects a combination of this effect and the helical propensities of the thiol containing amino acids.

**FIGURE 4:**
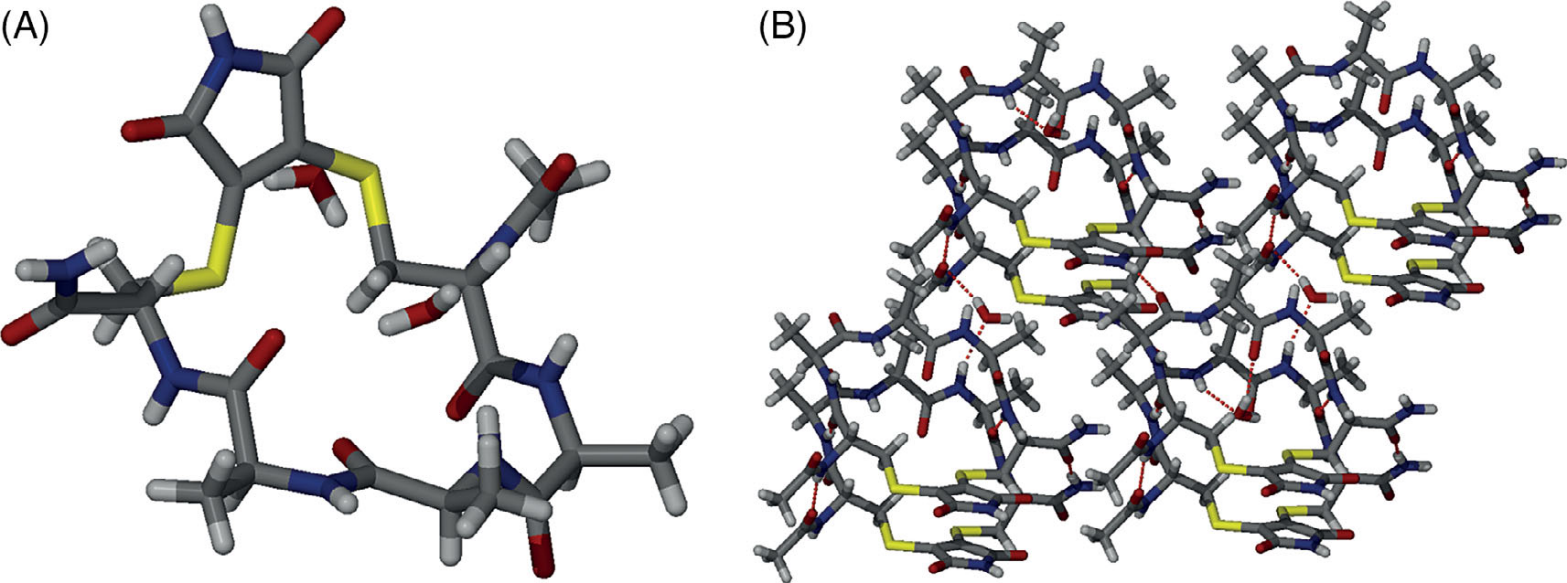
X‐ray structure analyses of peptide **2a**, (A) the peptide adopts a “flat” extended conformation in which there are no intramolecular hydrogen‐bonds and (B) packing of the peptide is mediated by intermacrocycle hydrogen‐bonding interactions, water‐mediated intermacrocycle hydrogen‐bonding and stacking of the maleimide unit

We also obtained a crystal structure of peptide **2a**. The conformation of the peptide in the solid state is of a “flat” extended nature (Figure 4) with packing mediated by intermacrocycle hydrogen‐bonding and stacking of the maleimide. The structure is reminiscent of a constrained peptide reported recently by Li and co‐workers^[^ ^50^ ^]^ and of the cyclic peptide nanotubes described originally by Ghadiri and co‐workers.^[^ ^51^, ^52^ ^]^ In the context of the current study, whilst **2a** has some helical propensity (25% in buffer and 41% in TFE), solid‐state hydrogen‐bonding and non‐covalent interactions of the staple highlight the complex effects on conformation that can occur through constraining a peptide.

Taken together, the conformational analyses confirm that the maleimide group is an effective bridging group to constrain peptides in an α‐helical conformation with *X*_1_ = L‐Cys and *X*_5_ = L‐*h*Cys the optimal combination of amino acids for maximal helicity as demonstrated for the model pentapeptide **2e**. The helical propensity of the amino acids themselves influences the helicity as does the constraint itself although both helix promoting properties and the non‐covalent behaviour of the staple should be considered. Similarly, the influence of proximal amino acids to the constraint should be considered; whilst these studies have been performed on a model pentapeptide, the constraint should serve simply to nucleate the helical conformation, hence steric and non‐covalent interaction with amino acids found inside the constrained sequence or distal to it, together with the length over which the nucleation site is required to propagate, should be considered.

## 4 CONCLUSIONS

In summary we have performed a comparative analysis on the α‐helix inducing properties of maleimide constraints in *i* to *i* + 4 bridged thiol containing pentapeptides. The study established that the rate of cyclisation is independent of the length of the constraint, indicating the size of the macrocycle is such that entropic disorder dominates the ring closure reaction. Most significantly we identified an optimal stapling combination of *X*_1_ = L‐Cys and *X*_5_ = L‐*h*Cys in the context of the model peptide Ac‐X_1_AAAX_5_‐NH_2_ and highlighted the ability of non‐covalent behaviour of the constraint to influence conformation. These studies should inform more effective use of maleimide based stapling reagents in future studies.

## DATA AVAILABILITY

CCDC‐1976850 contains additional information in cif format and can be obtained from the CCDC via www.ccdc.cam.ac.uk/structures/.

## AUTHOR CONTRIBUTIONS

A.J.W. conceived and designed the research programme, A.L.C., D.R.L. and M.W. designed studies and performed research, C.M.P. performed crystallographic analyses. The manuscript was written by M.W. and A.J.W. with contributions from all authors.

## CONFLICT OF INTERESTS

The authors have no conflicts to declare.

## Supporting information


**Appendix** **S1**: Supporting Information
