# Supplementary material for: Structural optimization of reversible dibromomaleimide peptide stapling
Source: Pept Sci (Hoboken). 2020 Mar 20;113(1):e24157. doi: 10.1002/pep2.24157 (PMC8650577; doi:10.1002/pep2.24157)
Supplement: Supplementary file 1 — Appendix S1: Supporting Information [file PEP2-113-e24157-s001.docx]

**Structural Optimization of Reversible Dibromomaleimide Peptide Stapling**

Ayanna Lindsey-Crosthwait,^a,b^ Diana Rodriguez-Lema,^a,b^ Martin Walko,^a,b^ Christopher M. Pask^a^ and Andrew J Wilson^a,b^

^a^School of Chemistry, University of Leeds, Leeds, LS2 9JT (UK) E-mail: a.j.wilson@leeds.ac.uk

^b^Astbury Centre for Structural Molecular Biology, University of Leeds, Leeds, LS2 9JT (UK).

***SUPPORTING INFORMATION***

**
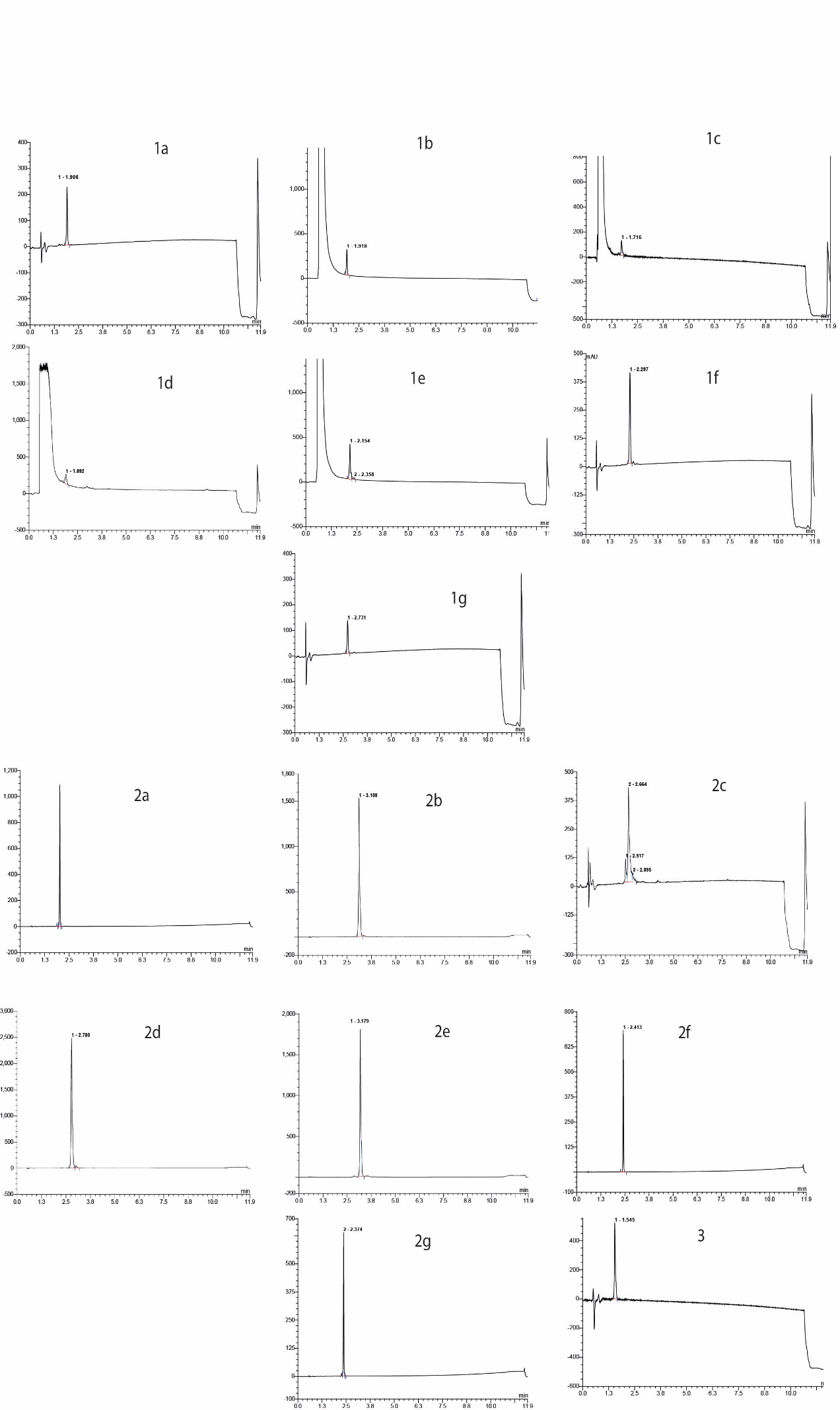
Figure S1**. HPLC traces for synthetic peptides

1b

1a

1b

1c

1d

1e

1f

1g

**Figure S2**. MS spectra for linear synthetic peptides

Except for M+H^+^ in most of the MS spectra additional peaks corresponding to M+Na^+^ and [M-NH_2_]^+^ can be observed

2a

2b

2c

2d

2e

2f

2g

3

**Figure S3**. MS spectra for cyclic synthetic peptides.

Except for M+H^+^ in most of the MS spectra additional peaks corresponding to [M-NH_2_]^+^ can be observed

| Empirical formula | C_21_H_33_N_7_O_10_S_2_ |
| --- | --- |
| Formula weight | 607.66 |
| Temperature/K | 120(2) |
| Crystal system | triclinic |
| Space group | P1 |
| a/Å | 4.9248(3) |
| b/Å | 12.6348(9) |
| c/Å | 12.6584(7) |
| α/° | 115.628(6) |
| β/° | 96.817(5) |
| γ/° | 95.044(6) |
| Volume/Å^3^ | 696.56(8) |
| Z | 1 |
| ρ_calc_g/cm^3^ | 1.449 |
| μ/mm^‑1^ | 2.311 |
| F(000) | 320.0 |
| Crystal size/mm^3^ | 0.14 × 0.04 × 0.02 |
| Radiation | CuKα (λ = 1.54184) |
| 2Θ range for data collection/° | 7.856 to 147.64 |
| Index ranges | -6 ≤ h ≤ 6, -15 ≤ k ≤ 15, -14 ≤ l ≤ 15 |
| Reflections collected | 7059 |
| Independent reflections | 4047 [R_int_ = 0.0447, R_sigma_ = 0.0602] |
| Data/restraints/parameters | 4047/3/413 |
| Goodness-of-fit on F^2^ | 1.073 |
| Final R indexes [I>=2σ (I)] | R_1_ = 0.0402, wR_2_ = 0.0945 |
| Final R indexes [all data] | R_1_ = 0.0448, wR_2_ = 0.0973 |
| Largest diff. peak/hole / e Å^-3^ | 0.34/-0.27 |
| Flack parameter | 0.03(2) |

**Table S1** Experimental data for the crystal structure determination of **2a**. CCDC-1976850 contains additional information in cif format and can be obtained from the CCDC via [www.ccdc.cam.ac.uk/structures/](http://www.ccdc.cam.ac.uk/structures/).
